# Draft genome sequences for two mucin-degrading bacteria isolated in Alabama

**DOI:** 10.1128/mra.00659-26

**Published:** 2026-09-21

**Authors:** Brice Gorman, Kristin Hobson, Melanie A. Higgins

**Affiliations:** 1 Department of Biological Sciences, The University of Alabama 8063, Tuscaloosa, Alabama, USA; Loyola University Chicago, Chicago, Illinois, USA

**Keywords:** mucin, environmental microbiology, *Pseudomonas*, *Bacillus*

## Abstract

Here, we describe the draft genome sequences of two mucin-degrading bacteria, *Pseudomonas kurunegalensis* strain chylan and *Bacillus thuringiensis* strain akera, isolated from The University of Alabama campus.

## ANNOUNCEMENT

Mucin is a heavily O-glycosylated protein degraded by specialized bacterial glycoside hydrolases (1, 2). However, most mucin-degradation pathways have been characterized only in human-associated bacteria (3). Here, we isolated mucin-degrading bacteria from soil as part of the Leaders Exploring Academic Possibilities summer program at the University of Alabama, expanding knowledge of mucin utilization beyond human-associated microbial communities.

The soil outside the Shelby Building at The University of Alabama in Tuscaloosa, Alabama (33.2114° N, 87.5401° W), was sampled using sterile swabs and streaked onto basal agar supplemented with 0.5% type II porcine gastric mucin (ThermoFisher Scientific) (4). Plates were incubated at 30°C for 48 h, and mucin-degrading colonies were restreaked onto fresh basal agar containing mucin and then Gram-stained to verify isolation. A single colony was grown overnight at 30°C and 150 RPM in 30 mL of Luria-Bertani broth until an OD_600_ of 0.6 was reached. Then 15 mL culture was harvested and centrifuged at 4,000 × *g* for 10 min. Resulting cell pellets were resuspended in 500 μL of Zymo 1× DNA/RNA Shield preservative. DNA extraction was performed by Plasmidsaurus (Louisville, Kentucky, USA) using the ZymoBIOMICS 96 MagBead DNA kit (Zymo Research). An amplification-free long-read sequencing library was constructed using kit v14 chemistry (Oxford Nanopore Technologies). Nanopore sequencing was performed utilizing R10.4.1 flow cells. The lowest-quality 5% of reads were removed using Filtlong v0.2.1 (5). Reads were downsampled to 100× coverage and assembled using Flye v2.9.1 (6), optimized for high-quality input, and Miniasm v. 0.3 (*Bacillus thuringiensis*) or Hifiasm v. August-2025 (*Pseudomonas kurunegalensis*) (7, 8). Assemblies were polished with Medaka v.1.6.1 (*Bacillus thuringiensis*) or Plassembler v1.8.0 (*Pseudomonas kurunegalensis*) (9, 10) and annotated using Bakta v1.11 (11). Assembly contigs were visualized with Bandage v0.8.1 (12), and genome completeness and contamination were assessed with CheckM v1.2.2 (13). Genome size and L50 were calculated using Biopython (14). Species identification was carried out using Mash v2.3 (15), Sourmash v4.6.1 (16), and CheckM v1.2.2 (13). Glycoside hydrolase genes were identified using dbCAN3 (17), with HMMER, DIAMOND, and dbCAN_sub. Default parameters were used for all software tools unless otherwise stated.

Genome sequences were deposited under the BioProject Number PRJNA1336800. The *P. kurunegalensis* strain chylan genome (BioSample SAMN52091390; TaxID: 485880) is a singular circular chromosome spanning 6,066,703 bp (Table 1). The *B. thuringiensis* strain akera genome (BioSample SAMN52091391; TaxID: 1428) comprises a 5,284,472 bp circular chromosome (contig 1) and 8 additional contigs. Contigs 2, 6, and 7 represent short chromosomal fragments. Contigs 4, 5, 8, and 9 share 78%–88% identity with *Planococcus donghaensis* strain DSM 22276 plasmid pPD67 (GenBank accession CP016544.1), while contig 3 is 77.7% identical to the *Bacillus toyonensis* strain P18 plasmid pBtP18 (GenBank accession CP064876.1), suggesting the strain contains two distinct plasmids. Both chromosomes were confirmed circular by overlapping long reads during assembly. Publicly available genome annotations are available via the National Center for Biotechnology Information (NCBI) Prokaryotic Genome Annotation Pipeline v6.8 (18).

**TABLE 1:** Genome assembly data information

Genome name	*Pseudomonas kurunegalensis* strain chylan	*Bacillus thuringiensis* strain akera
Size	6,066,703 bp	5,726,426 bp
Total sequenced bp	1,232,951,419	312,165,505
Total number of reads	218,682	74,929
Longest read	64,974	57,674
Genome coverage	102.4×	51.9×
N50 for assembly	6,066,703	5,151,858
Read N50	9,017 bp	6,851 bp
L50	1	1
Number of contigs	1	9
Completeness	99.87%	96.8%
Contamination score	1.3%	1.0%
G+C content	62%	35%
Number of annotated genes	5,524	5,893
Number of annotated glycoside hydrolases	68	74

## Data Availability

All data have been submitted to NCBI BioProject PRJNA1336800 with GenBank Accession numbers JBXYYF010000001.1 and JBRKPK010000001.1 to JBRKPK010000009.1 and BioSample numbers SAMN52091391 and SAMN57591111. The sequence read archive (SRA) numbers are SRR37153936 and SRR37153935.
